# “Z”‐Type Tilted Quasi‐One‐Dimensional Assembly of Actinide‐Embedded Coinage Metal Near‐Plane Superatoms and Their Optical Properties

**DOI:** 10.1002/advs.202206899

**Published:** 2023-01-25

**Authors:** Wanrong Huang, Famin Yu, Yu Zhu, Rui Wang, Jiarui Li, Sean Xiao‐An Zhang, Zhigang Wang

**Affiliations:** ^1^ Institute of Atomic and Molecular Physics Jilin University Changchun 130012 China; ^2^ State Key Laboratory of Supermolecular Structure and Materials Jilin University Changchun 130012 China; ^3^ International Center for Computational Method & Software College of Physics Jilin University Changchun 130012 China

**Keywords:** assembly, first‐principles, near‐plane superatom, UV‐Vis absorption spectra

## Abstract

In this work, a novel discovery that the coinage‐metal near‐plane superatoms (CM‐NPSs) formed by embedding actinide elements into the coinage metal rings can realize the “Z”‐type tilted quasi‐one‐dimensional (1D) direct assembly is reported. This success can be attributed to the strong bonding between the overlapping parts of adjacent superatomic motifs. First‐principles calculations reveal that the motifs maintain their geometric and electronic structures robustly during the assembly process. With the accumulation of motifs, the intensity of the absorption peak increases continuously in the ultraviolet‐visible (UV‐Vis) absorption spectra range of 300–450 nm, resulting in the hyperchromic effect, which is closely related to the degree of the participation of Th atoms. Furthermore, the absorption spectra show a continuously tunable feature in the 450–900 nm range, as the interlayer stacking pattern leads to a pronounced redshift. More importantly, the valence 5f‐shells of Th atoms have an increased contribution to the final orbitals of electronic transition, which demonstrates the advantages of the active high angular momentum electrons of actinide elements in spectral properties. These findings provide a valuable reference for the direct artificial assembly of near‐plane superatoms and optical properties of superatomic assemblies embedded with rare elements.

## Graphical abstract

A novel “Z”‐type tilted quasi‐1D assembly strategy suitable for near‐plane superatoms, which attributes to the complex electronic structure of the central atom is devised. With the increase of the number of motifs, the physicochemical properties of the monomer are maintained without changing the assembly mode. In addition, the regularity and mechanism of UV‐Vis absorption spectra are also understood.

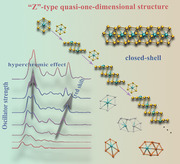

## 1 Introduction

Superatoms, with rich types and tunable properties, have been considered to show important prospects for assembling new functional materials and even become the driving force for their further development.^[^ ^1^ ^]^ Planar superatoms are distinguished from that of stereoscopic species, there is no component (i.e., z‐component) perpendicular to this plane for their molecular orbitals in the former. Therefore, the orbitals of planar superatoms have two‐dimensional (2D) ductility, which has a significant advantage in applications of tunable magnetism^[^ ^2^ ^]^ and aromaticity,^[^ ^3^ ^]^ the control of electronic excitation,^[^ ^4^ ^]^ and optical properties.^[^ ^5^ ^]^ They mainly include two types: pure plane and near plane. The former has identified observable differences from the three‐dimensional (3D) structure and has been extensively studied in the early stage.^[^ ^2^, ^3^ ^]^ The latter is a newer class, which always consists of a plane ring and a central atom that is not exactly on the same plane, but the formed structures still exhibit clear 2D ductility. However, during assembly, the 2D structures with ductility have greater difficulty in maintaining the stability than 3D ones. Especially for the coinage metal superatoms, even the 3D structures are difficult to directly assemble due to the weak bonding strength between metal atoms.^[^ ^6^ ^]^ Currently, theoretical and experimental studies show that the superatoms embedded by elements with high angular momentum electrons, such as actinides or lanthanides, can be stabilized and exhibit unique physicochemical properties under the influence of highly active valence electrons.^[^ ^7^ ^]^ These features are also suitable for near‐plane superatoms and provide an important prospect for the assembly of coated coinage metal superatoms.

In previous works, the properties and applications of planar superatoms have been extensively studied. For example, pure plane metallic superatoms embedded with transition atoms (Ti, V, Cr, Mn, Fe, etc.) exhibited high magnetic properties.^[^ ^2^, ^8^ ^]^ Embedded transition atoms (Ni, Pd, and Pt) also made these plane systems promising for catalytic applications.^[^ ^9^ ^]^ Besides, the Nb_2_@Au_6_ was found to use *σ* aromatism to stabilize the planar structure of metal rings.^[^ ^10^ ^]^ Actinide‐embedded near‐plane superatoms have prominent applications in surface‐enhanced Raman scattering.^[^ ^5^, ^11^ ^]^ In this context, how near‐plane superatoms with their unique properties can better assemble into novel materials has become a direction that requires a further effort. Nowadays, many assembly methods have been proposed,^[^ ^12^ ^]^ such as ligand passivation^[^ ^13^ ^]^ and adding atoms between monomers,^[^ ^14^ ^]^ etc.^[^ ^15^ ^]^ However, these approaches may introduce complex chemical bonds, causing changes in the geometric and electronic structures of superatomic motifs. Therefore, the development of a controllable assembly method that maintains the superatomic structural properties is still a crucial issue in the construction of functional materials.

In this work, our study shows that actinide‐embedded CM‐NPSs Th@Au_6_ can realize a “Z”‐type tilted quasi‐1D assembly pattern through the bonding of overlapping parts between adjacent monomers. Owing to the high stability of Th@Au_6_, the geometric and electronic structures of monomers can be kept intact during the assembly process. In addition, the UV‐Vis absorption spectra of assemblies exhibit regular changes with the increase of motifs, and represent new optical properties arising from the complex electronic transitions between orbitals in the motifs. These findings are expected to provide a new strategy for the direct assembly of near‐plane superatoms and offer prospects for exploiting of the optical properties of quasi‐1D assembled materials.

## 2 Results and Discussion

**Figure 1:**
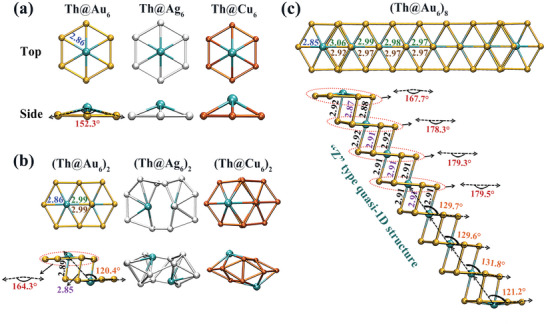
Geometric structures of Th@M_6_ (M = Au, Ag, and Cu) monomers and assembly. a) monomers, b) dimers and c) octamer. The top and side views of structures are given, and the corresponding bond lengths are noted. Since the assembly structures are symmetric along the center, all parameters are only marked on one side. The unit is Å. The same color represents the same parameter.

To achieve the goal of driven stabilization of assembly, we optimized and obtained the stable structures of Th@M_6_ (M = Au, Ag, and Cu), as shown in **Figure** **1**a. Both Th@Ag_6_ and Th@Cu_6_ also have the same superatomic electronic configuration as previously reported Th@Au_6_,^[^ ^5^ ^]^ namely $1S^{2}\;1P_{x}^{2}\;1P_{y}^{2}\;1D_{xy}^{2}\;1D_{x^{2}-y^{2}}^{2}$. Then, in Figure 1b, the monomers were assembled into the dimers to further explore the feasibility of assemblies. The results of structural optimization have shown that the geometry of Th@Au_6_ can be maintained after the assembly, while the Th@Ag_6_ and Th@Cu_6_ are severely damaged and clearly fused, respectively. This phenomenon can be explained by the stability of monomers. Compared with Th@Ag_6_ and Th@Cu_6_, Th@Au_6_ is the most stable structure and suitable for assembly (for details of monomer and dimer structures, see Figure S1–S7 and Table S1‐S3 in Supporting Information).

Based on the above analyses, we further realized the “Z”‐type quasi‐1D direct assembly with Th@Au_6_ as the motif. Moreover, we also proved that 2D assembly based on Th@Au_6_ is not feasible (Figure S8, Supporting Information). Figure 1c shows the optimized structure of octamer. Since the assembly structures are symmetric along the center, all parameters are marked on one side only. From the top view, the distance between the outermost Au that is not bonded to the adjacent motifs on both sides and the center Th is 2.85 Å (cf. monomer and dimer: 2.86 Å), while the bond lengths between Au that are bonded to the adjacent motifs and Th are 3.06 Å, 2.99 Å, 2.98 Å and 2.97 Å (cf. dimer: 2.99 Å), a gradually shorter trend is exhibited. The bond lengths of the second layer are also similar (2.92 Å and 2.97 Å). From the side view, the bond lengths (2.92 Å, 2.87 Å and 2.88 Å, cf. dimer: 2.89 Å and 2.85 Å) and the bond angles (∠Th–Th–Au = 121.2°, cf. dimer: 120.4°) between the outermost and the adjacent monomers changes slightly to maintain the near‐plane structure. While the bond lengths (2.92 Å and 2.91 Å) between the inner monomers are basically the same length, and the bond angles are basically around 130° (131.8°, 129.6°, and 129.7°). It is worth noting that the bond length between the four middle motifs has been stabilized to 2.91 Å, indicating that the middle region is already a quasi‐periodic structure and the system properties remain stable. This is also because both sides of the inner monomer are connected to adjacent motifs, so the forces on both sides are uniform. In addition, due to the stress of assembled structures, the monomer also tends to be planarized gradually, and the vertex angle ∠Au–Th–Au of the monomer changes from 152.3° for the monomer to 164.3° for the dimer. In the octamer, the vertex angle of the outermost monomers is 167.7°, while the vertex angles of the inner motif (178.3°, 179.3°, and 179.5°) are basically a pure plane structure. It is worth mentioning that the increase of motifs does not change the assembly mode, so the symmetry of assembly remains at C_2h_ (Figure S9, Supporting Information). This presents that assembly in the quasi‐1D direction has no essential effect on the symmetry of the structure.

Previous assembly strategy of “H”‐type nanotubes for planarized structures will complicate the bonding method between monomers, and even lead to the deformation of the structure.^[^ ^15^, ^16^ ^]^ The “Z”‐type quasi‐1D direct assembly strategy in this work is obviously different from previous reports. Here, the assembly is not only based on the combination of heavy metal actinide and coinage metals, but also simplifies the assembly of motifs. This also allows the six‐membered ring monomer involved in relevant self‐assembly to maintain its original structure and properties to a certain extent caused by quasi‐2D electron trajectory, in addition to the new properties given by the new electron trajectory in 3D space caused by such “Z”‐type assembly, even after self‐assembly. It is worth mentioning that the success of the assembly in this work is inseparable from the interaction of the central atom, which not only refers to the interaction with its own Au_6_ ring, but also the interaction with the adjacent Au atoms related to the formation of the assembly. In addition, the “Z”‐type assembly strategy is very similar to the *π* − *π* interaction of the benzene ring stacking,^[^ ^17^ ^]^ and is dominated by electrostatic interaction (Figure S5, Supporting Information), the magnitude of which depends on the overlap of electronic clouds between the superatoms (degree of offset between superatoms). This effect is also an important reason for the novel structural and electronic properties of superatoms in diverse dimensions. In particular, the connection between the rare elements rich in the high angular momentum of the electronic motion and the coinage metals will undoubtedly lead to stronger optoelectronic properties.

**Figure 2:**
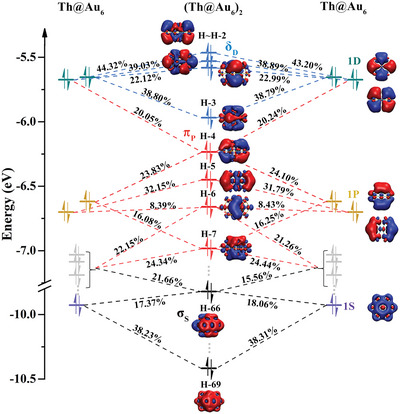
Orbital decomposition analysis of (Th@Au_6_)_2_. The energy levels of Th@Au_6_ and (Th@Au_6_)_2_, as well as the diagram of SAMOs. The H stands for HOMO.

To further explore the mechanism of assembly, we analyzed the molecular orbitals composition of (Th@Au_6_)_2_. As shown in **Figure** **2**, the fusion of the dimeric frontier orbitals all occurred in the overlapping part of motifs. Among them, the four orbitals H (the highest occupied molecular orbitals, HOMO) ≈H‐3 are all dimer *δ*_D_, the four orbitals H‐4 ≈ H‐7 are all *π*_P_, and the two orbitals H‐66 and H‐69 are *σ*_S_. It can be known that these dimeric orbitals are contributed by the 1S, 1P, and 1D superatomic molecular orbitals (SAMOs) of monomers, respectively. Here, superatomic clusters, as molecules, whose molecular orbitals are often called SAMOs. In (Th@Au_6_)_2_, the four orbitals of *δ*_D_ are completely contributed by the 1D orbitals of Th@Au_6_, and their contributions are all over 20%. The four orbitals of *π*_P_ are contributed by the 1P orbitals of motifs, partially doped with the contribution of 1D orbitals and non‐SAMOs. The *σ*_S_ is contributed by the 1S orbital of motifs and partially doped with the contribution of non‐SAMOs. It can be clearly seen that H‐69, H‐7, and H‐3 are the bonding orbitals of *σ*_S_, *π*_P,_ and *δ*_D_, respectively. The low position of the bonding orbital energy indicates that the system is prone to adhesion and stable assembly.

**Figure 3:**
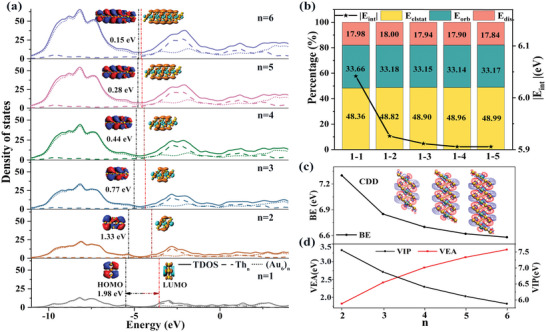
TheTDOS and interaction analyses of (Th@Au_6_)*_n_* (*n* = 1–6). a) The TDOS of (Th@Au_6_)*_n_* (*n* = 1–6). The long and short dashed lines represent the PDOS of Th and Au atoms, respectively. The figure shows the diagrams of HOMO and LUMO. The positions of HOMO and LUMO are marked by black and red dashed lines and give the HOMO‐LUMO gaps. b) The energy decomposition analysis (EDA) between the first monomer and the remaining fragments. c) The binding energy (BE) of (Th@Au_6_)*_n_* (*n* = 2–6) and some structured charge density difference (CDD). Because the CDD of assembly is the same, only the analysis results of *n* = 3‐5 are given. d) The vertical ionization potential (VIP) and vertical electron affinity (VEA) of (Th@Au_6_)*_n_* (*n* = 2–6). The abscissa is the number of motifs.

Next, we explored the electronic properties and interactions between the superatoms among the assembled structures. The density of states (DOS) can represent the number of electrons in the energy range, thus we calculated the total density of states (TDOS) and partial density of states (PDOS) of (Th@Au_6_)*_n_* (*n* = 1–6). The results showed that successively adding motifs to extend the structures in the 1D direction will cause the TDOS blue shift on the basis of numerical superimposition, but the overall curves trend does not change, as shown in **Figure** **3**a. This discloses that increasing the number of motifs does not change their electronic structures. Furthermore, the PDOS clearly shows that the Th atoms with high angular momentum electrons mainly contribute to orbitals with energy higher than the lowest unoccupied molecular orbitals (LUMO), while the Au atoms contribute to the orbitals with energy lower than LUMO, indicating that Th atoms have a huge contribution to the formation of the high angular momentum SAMOs. It is worth mentioning that as the number of motifs increases, the value of their HOMO gradually increases while LUMO gradually decreases. The gradual reduction of HOMO‐LUMO gaps (1.98, 1.33, 0.77, 0.44, 0.28, and 0.15 eV) represents an increase in the chemical activity of (Th@Au_6_)*_n_* (*n* = 1–6). According to previous studies, monomers with HOMO‐LUMO gaps larger than 1.5 eV tended to maintain their structural integrity and chemical properties during the assembly process,^[^ ^18^ ^]^ as fully demonstrated in this work. (The DOS of Th@Ag_6_ and Th@Cu_6_ dimers refer to Figure S7, Supporting Information).

Further, the frontier orbitals of assembled structures can form *δ* symmetry because of that they are mainly contributed by the 1D orbitals of monomers. The orbitals of assembly are connected in an orderly manner along the 1D direction, which is one of the reason for the stable assembly of Th@Au_6_. In addition, this work is different from the previous 1D chain‐like assembly approach that uses the magnetic coupling between spin‐polarized electrons to assemble the endohedral metal fullerene superatom U@C_28_.^[^ ^19^ ^]^ Since Th@Au_6_ is a closed‐shell singlet state, it can be effectively assembled without tuning the electron spin. Moreover, the HOMO and LUMO of U@C_28_ chain structures are located at the end of the chain, while those of Th@Au_6_ assembly structures are located on each monomer. This indicates that the SAMOs of each monomer contributes to the formation of assembly orbitals, and this combination method may be more conducive to structural stability.

To understand the source of stability of the assembled structures, energy decomposition analysis (EDA) was used for analyzing inter‐molecular interactions, in which the two selected fragments are the first monomer and the remaining part. As shown in Figure 3b, the *E*_elstat_, *E*_orb_, and *E*_dis_ of all structures accounted for ≈48%, 33%, and 18% to the total attractive interaction, indicating that the bonds between monomers are mainly connected by the ionic bonds. The assembly of monomers occurs when the attractive and repulsive interactions are balanced, which ultimately depends on *E*_int_ (Figure S5, Supporting Information). This has also been explained in previous reports.^[^ ^20^ ^]^ At the same time, |*E*_int_| gradually decreases as the number of monomers increases and tends to be stable. This further illustrates that the bonding way between the first monomer and the remaining fragments does not change as the number of monomers increases, and the structures gradually stabilize.

In order to investigate the binding ability and assembly mechanism of assembly structures, we calculated the binding energy (BE) and charge density difference (CDD) between the monomers of (Th@Au_6_)*_n_* (*n* = 2–6). As shown in Figure 3c, as the number of motifs increases, the BE between the monomers gradually decreases and becomes flat, indicating that the structures become more stable with the increase of motifs, which is consistent with the results of EDA. The CDD can intuitively obtain the electron flow direction after the interaction between the two fragments, reflecting the change of electron density during this process. It was found from the CDD that the charge accumulation areas are mainly distributed at the junction of Th and Au between the adjacent closed‐shell monomers, resulting in the formation of the bonds between the monomers. This is similar to the previous direct assembly of the closed‐shell superatoms W@Au_12_.^[^ ^21^ ^]^ Previous reports have also demonstrated the feasibility and structural stability of the superatomic assembly.^[^ ^22^ ^]^ The Voronoi deformation density charge distribution analysis also shows that both Th and Au atoms in the overlapping part lost electrons for bonding between Th@Au_6_ motifs (Tables S4, Supporting Information).

The results of the above analyses have shown that as the number of motifs increases, the relevant properties of the assembly expand orderly in the quasi‐1D direction, and the assembly mechanism has not changed significantly. Here, the characteristics of this assembly method are summarized briefly. First, the monomer should be a planarized structure with high stability. Second, the central atom has rich bonding properties, which can stabilize the planarized structures and link the monomers. Finally, the monomers are required to have the strong electronic local distribution ability, which will directly lead to a strong Pauli repulsion between monomers in face‐to‐face stacking, and repulsion and attraction are balanced in offset face‐to‐face stacking.

In addition, to further explore the electron gain and loss capability of assembly structures, we calculated the vertical electron affinity (VEA) and vertical ionization potential (VIP). As shown in Figure 3d, the results indicate that as the number of motifs increases, the VEA has a gradually increasing trend, while the VIP tends to decrease gradually. This further illustrates that the structures prefer to gain electrons during the assembly, suggesting that the assemblies have important applications in light excitation or light absorption. This also demonstrates the progressive increase in the oxidative properties of assembly, which is beneficial for future experimental characterization.

**Figure 4:**
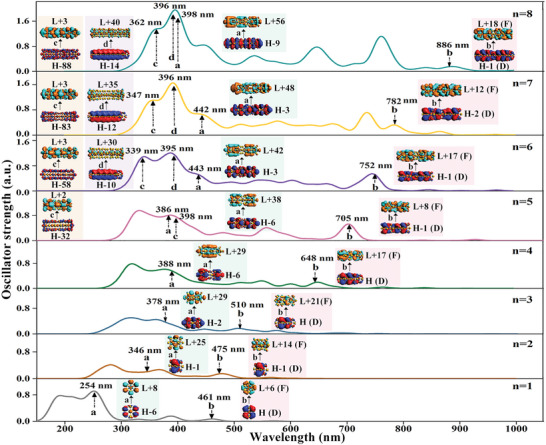
The UV‐Vis absorption spectra of (Th@Au_6_)*_n_* (*n* = 1–8). The typical transition source locations are marked with the same letter and color. On the right are the corresponding molecular orbitals of transition sources a‐d, respectively, where H and L stand for HOMO and LUMO. The D and F in the figure represent the D‐ and F‐SAMOs of Th@Au_6_, respectively. Peak width = 30.

Assembly is the foundation for gaining valuable functionality. For example, colloidal nanoparticles have been extensively studied in photophysics^[^ ^23^ ^]^ and gold nanoparticles in localized surface plasmon resonances.^[^ ^24^ ^]^ The UV‐Vis optical properties of the coinage metal systems are generally considered aspects.^[^ ^25^ ^]^ Here, based on the obtained assembled structures of CM‐NPSs, we analyzed the UV‐Vis spectra of outline the correlations between their structures and optical properties. As shown in **Figure** **4**, the spectra of (Th@Au_6_)*_n_* (*n* = 1–8) are mainly located in the wavelength range of 150–1000 nm. It can be clearly seen that the significant absorption peaks are mainly concentrated in the short wavelength region from 300 to 450 nm and the long wavelength region from 450 to 900 nm.

In the short wavelength range, it can be observed that the intensity of the absorption peak increases continuously as the motif increases, resulting in the hyperchromic effect. Specifically, when *n* = 2–5, the intensity of the first peak is higher than that of the second peak. At *n* = 6–8, the second peak gradually becomes sharper and higher than the first peak. Meanwhile, there is a tendency for the two peaks to gradually merge, resulting in a shoulder peak around 440 nm. To further explore the reason for this phenomenon, the dominant electronic transition orbitals of the absorption peaks were investigated, as shown in Figure 4. Because the peaks in the spectra are formed by fitting oscillator intensities, larger oscillator intensities are not necessarily at the highest position of the peak. It can be found that the electronic transition sources of the absorption peaks at the short wavelengths all have components similar to those of monomers (cf. electronic transition source a in Figure 4). Furthermore, as the increase of motifs, the contribution of the absorption peaks becomes more complex. Among them, different new electronic transition sources (cf. electronic transition sources c and d) appear when *n* = 5 and 6, respectively. When *n* = 8, the electronic transition sources gradually merged, which also directly leads to the coalescence and sharpening of the absorption peaks. This suggests that the spectral properties in the short wavelength region are caused by the accumulation of motifs, and the absorption bands formed by different electronic transitions will be induced in the short wavelength range, which cannot be separated from the superposition of interactions between the systems. It should be noted that the contribution of 5f‐shell in Th elements increases to vary degrees for the electronic transition. As shown in **Table** **1** and Figure 4, the electronic transition from the Au ring to the whole (electronic transition source c), the electron transition from the bonding orbital of the overall D‐SAMOs to the central Th atom (electronic transition source d), the contribution of 5f‐shell of Th atoms up to ≈90%. The appearance of the electronic transition source d is responsible for the significant enhancement of the absorption peaks, indicating that the participation of Th atoms is the essence of the hyperchromic effect. To visualize the contribution of Th atoms more clearly, the orbitals of the transition sources a‐d were scaled up equally in Figure S10, Supporting Information.

**Table 1:** Excitation wavelength, electron transition orbitals, the contribution of corresponding Th atoms and the contribution of Th valence shell of typical transitions between SAMOs in the UV‐Vis spectra of (Th@Au_6_)*_n_* (*n* = 1–8). Contributions of Th atoms that account for less than 1% are not included in the statistics

motifs		Λ [nm]	Transitions (Th contribution/%)	Contribution of Th atomic valence shell/%
*n* = 1	a	253.60	H‐6 (1.22) → L+8 (44.41)	6d: 1.22 → 5f: 44.41
	b	461.43	H (14.68) → L+6 (79.54)	6d: 14.68 → 5f: 79.54
*n* = 2	a	345.88	H‐1 (16.22) → L+25 (42.91)	5f: 1.01, 6d: 13.35, 7p: 1.86 → 5f: 15.03, 6d: 25.74, 7p: 2.14
	b	475.26	H‐1 (16.22) → L+14 (94.01)	5f: 1.01, 6d: 13.35, 7p: 1.86 → 5f: 91.05, 6d: 1.41, 7p: 1.55
*n* = 3	a	377.73	H‐2 (21.62) → L+29 (54.73)	7s: 1.34, 6d: 19.20, 7p: 1.08 →7s: 8.89, 5f: 35.44, 6d: 4.04, 7p: 6.27
	b	509.58	H (16.16) → L+21 (85.92)	5f: 1.48, 6d: 13.66, 7p: 1.02 → 5f: 79.09, 6d: 6.83
*n* = 4	a	387.71	H‐6 (10.98) → L+29 (57.61)	7s: 1.03, 6d: 9.95 →7s: 30.08, 5f: 8.04, 6d: 14.38, 7p: 5.11
	b	648.30	H (13.21) → L+17 (69.49)	5f: 2.04, 6d: 11.17 →5f: 53.93, 6d: 15.56
*n* = 5	a	386.28	H‐6 (9.97) → L+38 (47.36)	6d: 9.97 →7s: 26.34, 5f: 10.95, 6d: 10.07
	b	705.20	H‐1 (16.54) → L+8 (50.15)	5f: 1.34, 6d: 15.20 →5f: 10.84, 6d: 37.56, 7p: 1.75
	c	397.60	H‐32 (2.77) → L+2 (35.81)	7s: 2.77 →5f: 1.21, 6d: 34.60
*n* = 6	a	442.74	H‐3 (16.79) → L+42 (56.71)	6d: 16.79 →7s: 17.31, 5f: 26.98, 6d: 8.17, 7p:4.25
	b	752.13	H‐1 (15.67) → L+17 (59.66)	5f: 1.72, 6d: 13.95 →5f: 27.60, 6d: 32.06
	c	339.20	H‐58 (0.00) → L+3 (34.30)	0.00 →5f: 2.32, 6d: 31.98
	d	395.06	H‐10 (19.85) → L+30 (90.02)	6d: 19.85 →5f: 88.44, 6d: 1.58
*n* = 7	a	442.16	H‐3 (19.18) → L+48 (73.75)	6d: 19.18 →7s: 6.00, 5f: 59.16, 6d: 5.54, 7p:3.05
	b	782.41	H‐2 (14.63) → L+12 (49.85)	5f: 1.03, 6d: 13.60 →5f: 13.09, 6d: 36.76
	c	347.08	H‐83 (0.00) → L+3 (31.37)	0.00 →7s: 1.44, 5f: 1.53, 6d: 28.40
	d	395.59	H‐12 (21.80) → L+35 (90.27)	6d: 21.80 →5f: 88.00, 6d: 2.27
*n* = 8	a	397.67	H‐9 (21.84) → L+56 (58.36)	6d: 21.84 →7s: 15.81, 5f: 33.73, 6d: 6.47, 7p:2.35
	b	885.96	H‐1 (13.00) → L+18 (51.44)	5f: 1.43, 6d: 11.57 →5f: 15.23, 6d: 36.21
	c	361.52	H‐88 (0.00) → L+3 (27.11)	0.00 →7s: 2.08, 5f: 1.55, 6d: 23.48
	d	396.22	H‐14 (21.17) → L+40 (87.04)	6d: 21.17 →5f: 85.49, 6d: 1.55

However, different from the short wavelength, in the long wavelength range, there are absorption peaks of the same electronic transition source (electronic transition source b), which are all the electronic transitions of D‐SAMOs to the F‐SAMOs of Th@Au_6_. The interlayer stacking mode leads to obvious redshift, which shows the continuously tunable features of the spectrum. This is similar to the spectral law of the Au_13_ cluster aggregates.^[^ ^26^ ^]^ Importantly, doping with Th atoms would give the system special optical properties beyond those of pure gold nanoparticles in terms of electron transitions. It can be seen from Table 1, the contribution of s, p, d, and f atomic orbitals in 5f‐shell of Th atoms to the electronic transition orbital's changes from about 15% to more than 50%, indicating that these electronic transition orbitals are transitions from orbitals dominated by Au atoms to orbitals dominated by Th atoms (electronic transition source b). The PDOS analysis suggests that this is explained by the fact that most of the electrons of Au participate in occupied SAMOs, while the electrons of Th are involved in unoccupied high angular momentum SAMOs. It is also noteworthy that as the number of motifs increases, their absorption peaks show a non‐monotonic increase. Specifically, for *n* = 1–5, their peaks gradually increase and the sources were single, while for *n* = 6–8, their peaks gradually decreased and the sources become more complex. The reason for the decrease of the peak value is due to the electronic transition sources having a changing trend when *n* = 6. Peak splitting is evident at *n* = 7, and the source of the highest absorption peak is completely transformed into a new electronic transition source (The detailed electronic transition orbitals information statistics are in Table S5, Supporting Information). In addition, previous studies have shown that the interlayer stacking patterns play an important role in controlling optical properties, among others.^[^ ^27^ ^]^ For example, the way of stacking strongly affects the interlayer coupling strength, thus significantly changing their band structures, and is prone to various quantum phase transitions.^[^ ^28^ ^]^ The difference in stacking mode is also the key reason for the difference in layer‐dependent evolution trends in the center energies of feature peaks in the optical conductivity spectra.^[^ ^29^ ^]^ In this work, this offset face‐to‐face stacking also makes the electrons more delocalized between the layers, so the electron correlation between the motifs is tighter. This results in the superposition of interlayer interactions exhibiting spectral redshifts at long wavelengths.

Based on the above results, both in the short wavelength and long wavelength regions, the characteristic peaks lead to spectral complexity with the increase of motifs, which is mainly attributed to the complex different electronic transitions between assemblies. Meanwhile, the characteristic peaks in the short wavelength and long wavelength regions also reflect distinct spectral characteristics that help analyze the information about the structure of assemblies themselves and the interactions between superatoms.^[^ ^30^ ^]^ More importantly, as indicated above for the gold clusters, the participation of Th atoms makes their contribution to the SAMOs significantly increased during the electronic transition process, indicating that the actinide‐embedded assembled materials have exotic properties in terms of optical properties. These results have important applications and reference values for the luminescence properties of 1D assembled materials. In addition, the application of superatoms in surface‐enhanced Raman scattering is also promising, especially for actinide‐embedded 2D and 3D superatoms.^[^ ^5^, ^11^, ^31^ ^]^ In the future, we will further explore the mechanism and advantages of such “Z”‐type tilted quasi‐1D assembly in the application of surface‐enhanced Raman scattering.

## 3 Conclusion

In summary, we investigated the “Z”‐type tilted quasi‐1D structures assembled by actinide‐embedded CM‐NPSs Th@Au_6_ and their UV‐Vis absorption spectra using the first‐principles calculations. The results demonstrat that the high stability of the Th@Au_6_ enables it to maintain structural properties during the quasi‐1D assembly process. Its assembly pattern is similar to the offset face‐to‐face stacking of benzene rings, which is mainly dominated by the accumulation of charges between adjacent monomers, leading to the bonding of overlapping parts. Not only that, the orbital decomposition analysis presents that the energy of bonding orbitals is lower, and the system is easy to stick and assemble stably. In addition, the spectra of the assemblies in different regions show regular changes as the number of superatoms increases. In the short wavelength range, the participation of Th atoms gives rise to the hyperchromic effect. In the long wavelength range, the redshift of the spectra is caused by the stacking mode, and the absorption peak sources are all electronic transition from the D‐SAMOs to F‐SAMOs, showing a continuously tunable feature. These spectral properties will be helpful for experimental characterization and future spectral exploration of the assembly of actinide‐embedded near‐plane structures. This work will enrich the assembly strategy for 2D superatoms, which further lay the foundation for better control of superatomic functions, and provide a premising for exploring the optical properties of the quasi‐1D assembly.

## 4 Experimental Section

These calculations were based on the density functional theory with the third‐generation empirical dispersion correction (DFT‐D3) method, generalized gradient approximation (GGA) Becke–Perdew (BP86) exchange‐correlation functional was also adopted throughout this work.^[^ ^32^ ^]^ A triple‐*ζ* with polarization functions (TZP) Slater basis set was employed. The frozen core for Th is [1s^2^–4f^14^]. The frozen core for Au, Ag, and Cu are [1s^2^‐4d^10^], [1s^2^‐3d^10^] and [1s^2^‐2p^6^], respectively. The zero‐order regular approximations (ZORA) take into account the relativistic correction of heavy elements.^[^ ^33^ ^]^ The geometry optimization and other analysis calculations were performed using the Amsterdam density functional (ADF) package.^[^ ^34^ ^]^ The orbital decomposition analysis and CDD were also calculated at BP86/LANL2DZ level using the Gaussian 09 program and Multiwfn analyses.^[^ ^35^ ^]^ The optimized structure is also verified on Gaussian 09.

We only focus on the binding ability between fragments, so BE formula:

(1) $$\begin{array}{ccc} & & \mathrm{BE}\left[{\left(\mathrm{Th}@{\mathrm{Au}}_{6}\right)}_{n}]\;=\;[E\left(\mathrm{Th}@{\mathrm{Au}}_{6}\right)+\cdots +E\left(\mathrm{Th}@{\mathrm{Au}}_{6}\right)-E{\left(\mathrm{Th}@{\mathrm{Au}}_{6}\right)}_{n}\right]/\\ & & \;\left(n-1\right) \end{array}$$

The EDA proposed by Morokuma, Ziegler, and Rauk is the most widely used quantitative analysis method.^[^ ^36^ ^]^ The total interaction energy of energy decomposition can be divided into four parts, namely Pauli repulsion, electrostatic interaction, orbital interaction, and dispersion interaction:

(2) $$E_{\mathrm{int}}=E_{\mathrm{pauli}}+E_{\mathrm{elstat}}+E_{\mathrm{orb}}+E_{\mathrm{dis}}$$

The first term is repulsive interaction, and the last three terms are attractive interaction. The values of VIP and VEA are defined as follows:

(3) $$E_{\mathrm{VIP}}=E^{+}\left(\mathrm{sp}\right)-E^{0}\left(\mathrm{opt}\right)$$

(4) $$E_{\mathrm{VEA}}=E^{0}\;\left(\mathrm{opt}\right)-E^{-}\left(\mathrm{sp}\right)$$

*E* ^0^(opt), *E* ^+^(sp) and *E* ^−^(sp) are the total energy of the relaxed neutral system and the energy of the unrelaxed cation and anion molecule, respectively.

## Conflict of Interest

The authors declare no conflict of interest.

## Supporting information

Supporting Information

## Data Availability

The data that support the findings of this study are available from the corresponding author upon reasonable request.
