# Supplementary material for: “Z”‐Type Tilted Quasi‐One‐Dimensional Assembly of Actinide‐Embedded Coinage Metal Near‐Plane Superatoms and Their Optical Properties
Source: Adv Sci (Weinh). 2023 Jan 25;10(9):2206899. doi: 10.1002/advs.202206899 (PMC10037954; doi:10.1002/advs.202206899)
Supplement: Supplementary file 1 — Supporting Information [file ADVS-10-2206899-s001.pdf]

Supporting Information

“Z”-type tilted quasi-one-dimensional assembly of actinide-embedded coinage metal near-plane superatoms and their optical properties

Wanrong Huang, Famin Yu, Yu Zhu, Rui Wang, Jiarui Li, Sean Xiao-An Zhang and Zhigang Wang**

Wanrong Huang, Famin Yu, Yu Zhu, Rui Wang, Jiarui Li, Zhigang Wang
Institute of Atomic and Molecular Physics
Jilin University
Changchun 130012, China
E-mail: wangzg@jlu.edu.cn (Zhigang Wang)

Sean Xiao-An Zhang
State Key Laboratory of Supermolecular Structure and Materials,
Jilin University,
Changchun 130012, China
E-mail: seanzhang@jlu.edu.cn (Sean Xiao-An Zhang)

Zhigang Wang
International Center for Computational Method & Software
College of Physics
Jilin University
Changchun 130012, China

Contents

Figure S1. Energy levels of Th@M_6.

Figure S2. The structure details of Th@M_6 (M=Au, Ag, Cu).

Figure S3. The charge density difference (CDD) of Th@M_6.

Figure S4. The energy decomposition analysis (EDA) and redox reaction analysis of Th@M_6.

Figure S5. The interaction analyses of (Th@Au_6)$_2$.

Figure S6. The trimers and tetramers assembly of Th@Ag_6 and Th@Cu_6.

Figure S7. The density of states (DOS) of (Th@Ag_6)$_n$ (n= 1, 2) and (Th@Cu_6)$_n$ (n= 1, 2).

Figure S8. The two-dimensional assembly of (Th@Au_6)$_n$ (n=2-4).

Figure S9. The structural information of (Th@Au_6)$_n$ (n=1-8).

Figure S10. The transition source a-d of (Th@Au_6)$_n$ (n = 1-8) in the ultraviolet-visible (UV-Vis) absorption spectra.

Table S1. Interaction analyses of Th@M_6.

Table S2. The Wiberg bond order of Th-M bonds.

Tables S3. The Voronoi deformation density (VDD) charge distribution analyses of Th@M_6.

Tables S4. The Voronoi deformation density (VDD) charge distribution analyses of (Th@Au_6)$_n$ (n= 2-6).

Table S5. The UV-Vis spectral of (Th@Au_6)$_n$ (n=1-8).

Tables S6-S17. The optimized structural coordinates of (Th@Au_6)$_n$ (n=1-8).

Reference

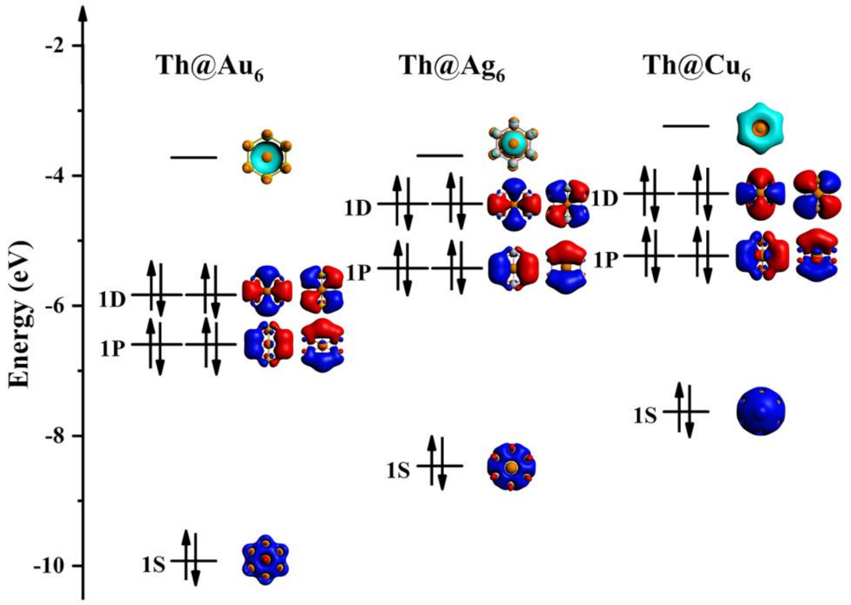

Figure S1. Energy levels of Th@M_6 (M=Au, Ag, Cu). The 1S, 1P and 1D superatomic molecular orbitals (SAMOs) are listed.

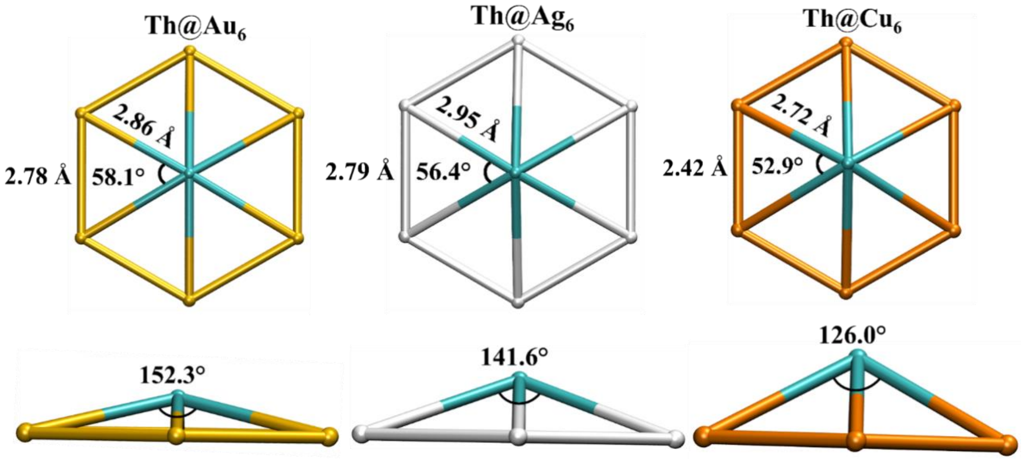

Figure S2. Optimized structures of Th@M_6. The top view shows the length of Th-M bond, the length of M-M bond, and the angle between adjacent coinage metal atoms and the Th atom. The side view shows the angle between diagonal coinage metal atoms and the Th atom.

Due to the C_{6v} symmetry, their M-Th-M bond angles are the same, meanwhile their Th-M and M-M bonds are equivalent, respectively. The bond angles Au-Th-Au, Ag-Th-Ag and Cu-Th-Cu formed by adjacent coinage metal atoms and the central atom are 58.1°, 56.4° and 52.9°, respectively. The bond angles formed by the diagonal coinage metal atoms and the central atom are 152.3°, 141.6° and 126.0°, respectively. The bond lengths of Th-Au, Th-Ag and Th-Cu are 2.86, 2.95 and 2.72 angstrom, respectively. The bond lengths of Au-Au, Ag-Ag and Cu-Cu are

2.78, 2.79 and 2.42 angstrom. We found that the bond angles gradually decrease with the change of coinage metal atoms from Au to Cu, which also makes the overall structure gradually appear non-planar. In addition, when the coinage metal atoms change from Au to Cu, the law of Th-M is: Th-Ag>Th-Au>Th-Cu, the law of M-M is: Ag-Ag>Au-Au>Cu-Cu. The sum of Th-M covalent radii: Th-Au> Th-Ag> Th-Cu, which does not conform to the bond length laws obtained in this work. The law of the bond lengths is mainly due to the relativistic effect of heavy elements, which is consistent with previous knowledge.[1]

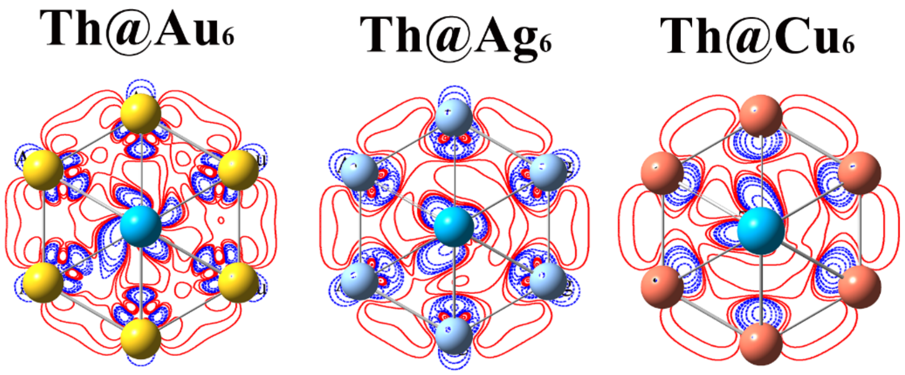

Figure S3. The charge density difference (CDD) of Th@M_6. Red solid lines and blue dashed lines represent the electron aggregation and dissipation. Dark blue, yellow, light blue and orange spheres represent Th, Au, Ag and Cu elements, respectively.

The charge density difference (CDD) of three structures were further analyzed. It was found that electrons gathered at the interconnection of each atom, indicating that the atoms were bonded.

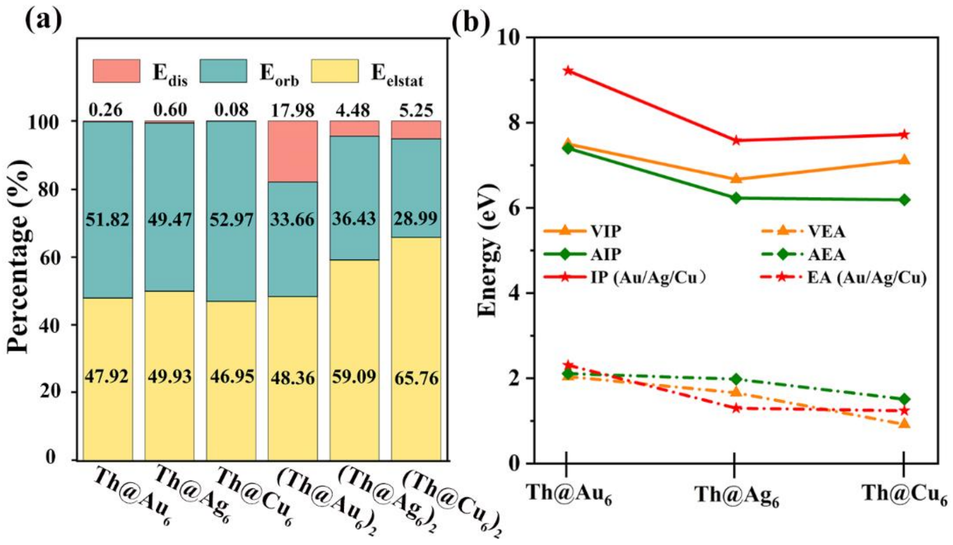

Figure S4. The energy decomposition analysis (EDA) and redox reaction analysis. (a) The EDA of Th@M_6 and (Th@M_6)$_2$. For monomers, two selected fragments of EDA are the coinage metal rings and the central atom. For dimers, it is between monomers. Yellow, green and red areas represent the percentage of the electrostatic interaction (E_{elstat}), orbital interaction (E_{orb}) and dispersive interaction (E_{dis}) to the total attractive interaction, respectively. (b) The redox reaction analysis of Th@M_6. Yellow and green solid lines and dashed lines represent the vertical ionization potential (VIP) and adiabatic ionization potential (AIP), the vertical electron affinity (VEA) and adiabatic electron affinity (AEA), respectively. The red solid line and dashed line represent the ionization potential (IP) and electron affinity (EA) of the coinage metal atoms, respectively.

In order to explore the interactions between the coinage metal rings and the central atom, energy decomposition analysis (EDA) was performed. Two selected fragments for EDA are the coinage metal rings and the central atom. For Th@Au_6 and Th@Cu_6, the orbital interaction (E_{orb}) accounts for more than 50% of the total attractive interaction energy, which shows that the E_{orb} is dominant. Therefore, the bonds between the Au_6 and Cu_6 rings and the central atom tend to be covalent. However, for Th@Ag_6, the electrostatic interaction (E_{elstat}) is slightly larger than the E_{orb}, the bonds between the Ag_6 ring and Th tend to be ionic. The monomers are divided into two fragments of the dimers. The percentages of E_{elstat} to the total attraction interaction in (Th@Au_6)$_2$, (Th@Ag_6)$_2$ and (Th@Cu_6)$_2$ are 48.36%, 59.09% and 65.76%, respectively. The results reveal that the interactions between the monomers are dominated by E_{elstat}, indicating that are mainly connected by ionic bond.

We further calculated vertical ionization potential (VIP), adiabatic ionization potential (AIP), vertical electron affinity (VEA) and adiabatic electron affinity (AEA) of three assembled structures. It is found that the law of VIP with the change of the shell atom from Au to Cu is: Th@Au_6>Th@Cu_6>Th@Ag_6, and the law of VEA and AEA is: Th@Au_6>Th@Ag_6>Th@Cu_6. The greater the ionization energy, the less likely it is to lose electrons. The greater the affinity energy, the easier it is to get electrons. It can be seen that Th@Au_6 can easily obtain electrons and exhibit oxidizing properties. Because the geometric configuration is deformed when calculating the AIP, which makes the AIP meaningless, we will not go into too much detail about it in this work. Previous reports indicate that the law of IP in coinage metal atoms was found to be Au>Cu>Ag[2], and the law of EA is Au>Ag>Cu[3]. It is preliminarily concluded that when changing the coinage metal atoms of the outer shell, it is the law of the atoms themselves that determines that different.

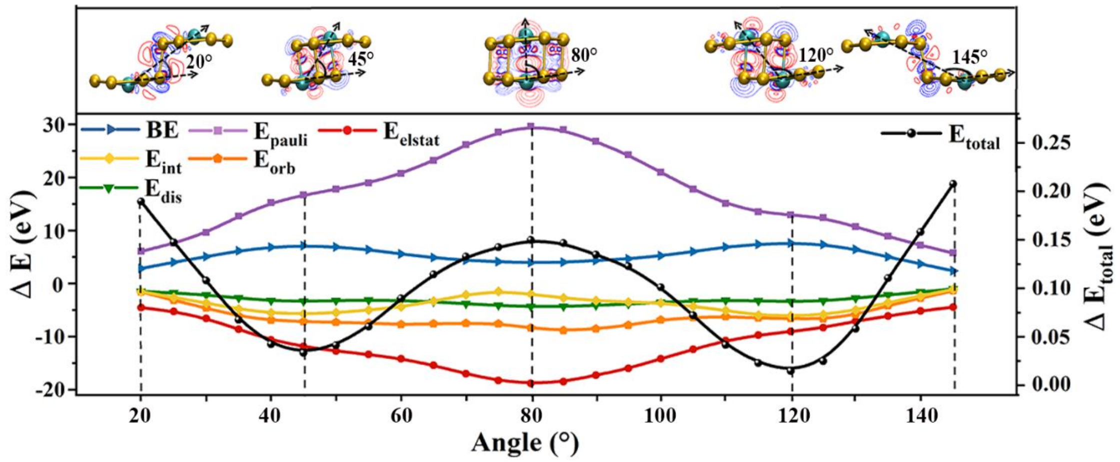

Figure S5. The CDD analysis at five special structures and interaction analyses with the change of ∠Th-Th-Au (marked as α). The structure with α=120° is the most stable structure, α=80° is the face-to-face structure of two monomers, and α=45° is the sub-stable structure. The α=20° and α=145° are the structures with the farther distance between monomers. The red and blue solid lines in CDD indicate electronic aggregation and dissipation, respectively.

To gain insight into the formation mechanism of this "Z"-type offset stacked assembled structure $(Th@Au_6)_2$, we investigated the interaction and potential energy curve changes during monomer slip process. The change of ∠Th-Th-Au (marked as α) was used to indicate the degree of slippage between monomers. As showed in Figure S5, it can be seen from the potential energy curve that a local minimum appears as α gradually increases to 45°. When the angle gradually increased to 80°, a saddle point appeared, and continued to increase to 120° to reach the lowest point of energy. The binding energy (BE) between monomers at each point is consistent with the conclusion of the potential energy curve. Both confirmed that the structure with the α of 120° is the most stable. Then, five typical structures on the potential energy curve are selected for the CDD analyses. It is found that the charge accumulation area is mainly distributed at the junction of Th and Au between adjacent monomers, resulting in the formation of bonds between the monomers. This indicates that the assembly of monomers mainly involves the charge accumulation between them. To further explore the properties of bonds between monomers, the law of EDA is explored with the changes of α. The results disclose that the value of E_{elstat} is always the largest in the attractive interaction, indicating that the bond between monomers is mainly connected by the ionic bond. But from the most stable structural point of view, monomers assembly occurs when they are balanced by the interaction of attraction and repulsion, which

ultimately depends on E_{int}. This has also been explained in previous reports[4]. In the structure of α=120°, E_{int} is the largest, which further proves that the structure is the most stable. This is basically consistent with the results of the potential energy curve and the BE.

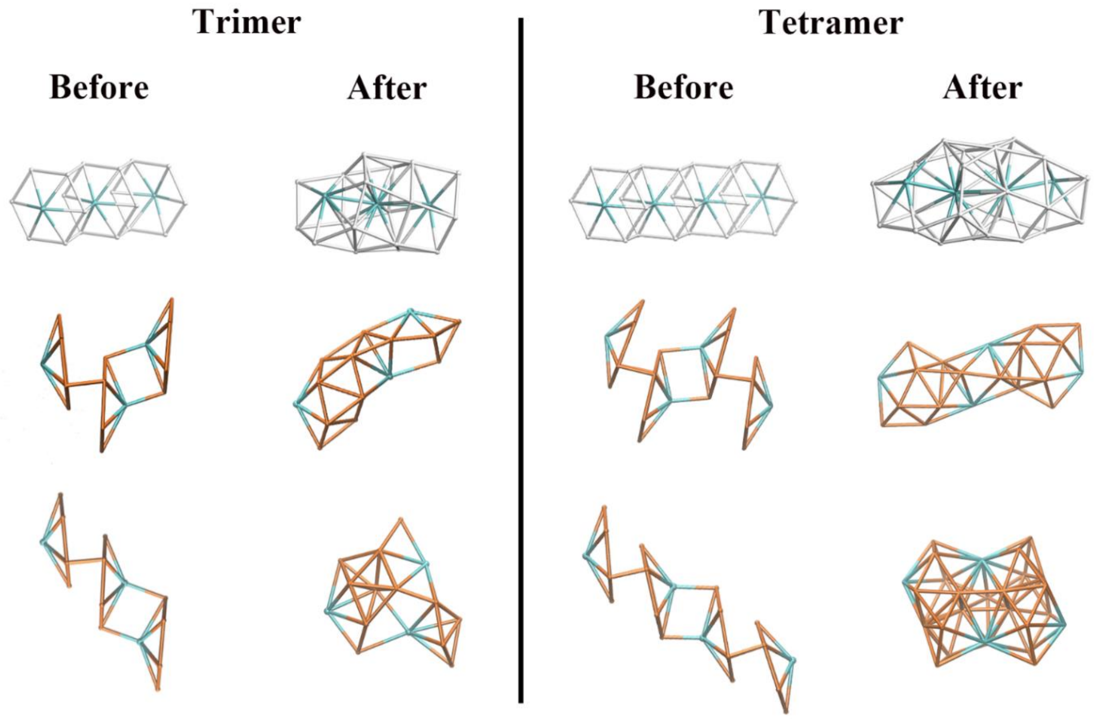

Figure S6. Trimers and tetramers of Th@Ag_6 and Th@Cu_6. The left half and right half are trimer and tetramer structures, respectively. Among them, the left side and right side are the initial and final structures, respectively. Th@Cu_6 has two assembly methods.

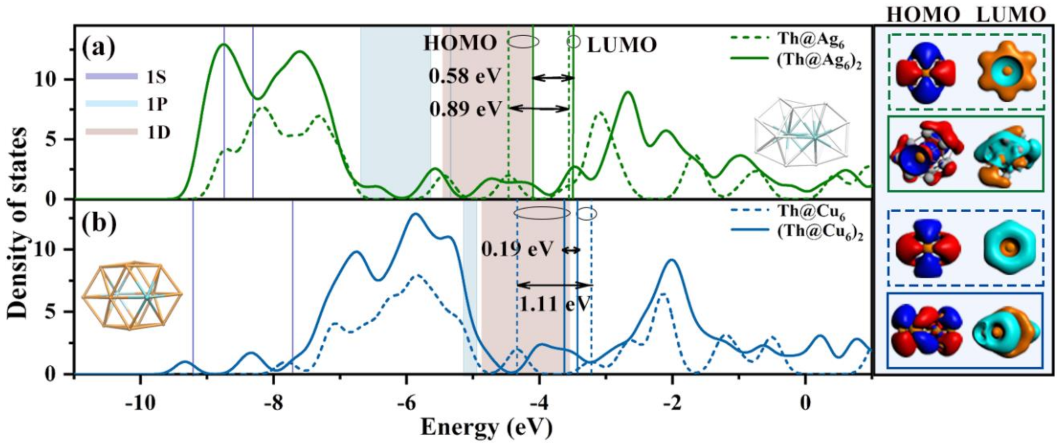

Figure S7. The density of states (DOS) of $(Th@Ag_6)_n$ and $(Th@Cu_6)_n$ (n= 1, 2). (a) The DOS of $(Th@Ag_6)_n$. (b) The DOS of $(Th@Cu_6)_n$. The dotted and solid lines represent the DOS of monomers and dimers, respectively. The diagrams of monomer and dimer highest occupied molecular orbital (HOMO) and lowest unoccupied molecular orbital (LUMO) are given on the right. The position of HOMO and LUMO are displayed and the H-L gap is marked. The purple, blue and pink areas indicate the locations of the SAMOs 1S, 1P and 1D, respectively.

From the density of states (DOS) of Th@Ag_6 and Th@Cu_6 monomers and dimers, it is found that after Th@Cu_6 is assembled into dimers, the trend of DOS and the position of the main peak remain basically unchanged. The position of the main peak has shifted due to the distortion of the Th@Ag_6 dimer structure. It is worth note that both HOMO and LUMO of $(Th@Cu_6)_2$ can form δ symmetry, while the frontier molecular orbitals of $(Th@Ag_6)_2$ cannot be formed, indicating that the structure of $(Th@Ag_6)_2$ is asymmetry. This further proves that Th@Ag_6 did not maintain the electronic properties during the assembly process.

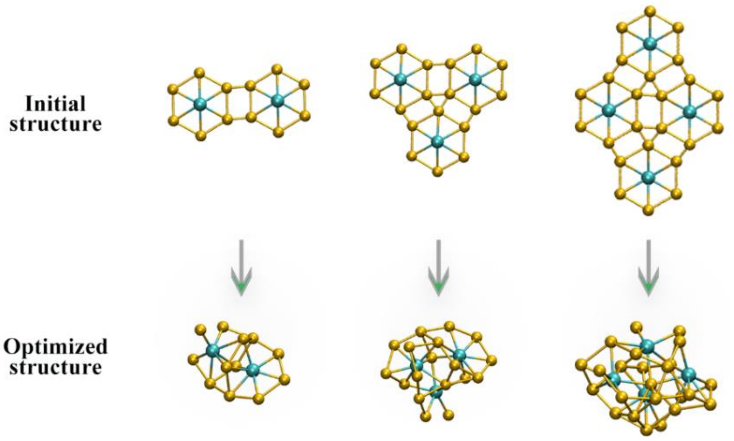

Figure S8. The initial and optimized structures of the two-dimensional assembly of $(Th@Au_6)_n$ (n=2-4).

As shown in Figure S8, our calculated results have presented that the two-dimensional assembly of Th@Au_6 as motifs seems invalid. On the one hand, in the two-dimensional extension process, the interactions of the central Th elements and Au elements are absent, resulting in the inability to support the structure maintaining the two-dimensional state. Thus, the phenomenon of deformation and fusion occurs. On the other hand, since Th@Au_6 itself belongs to near-plane superatom rather than that of pure plane, it is also difficult to maintain the structural properties of the monomers during the two-dimensional assembly process and tends to form aggregate structures.

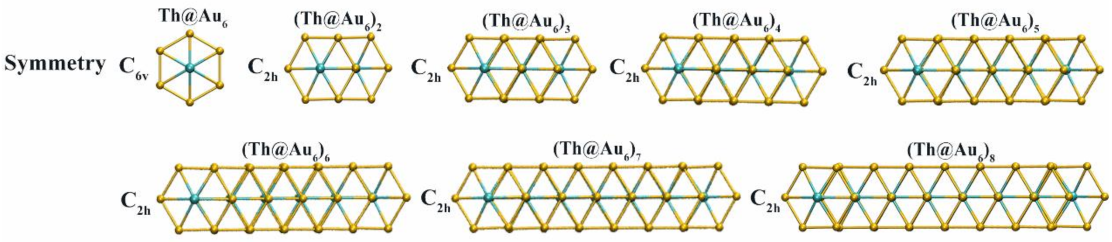

Figure S9. The structural information of $(Th@Au_6)_n$ (n=1-8).

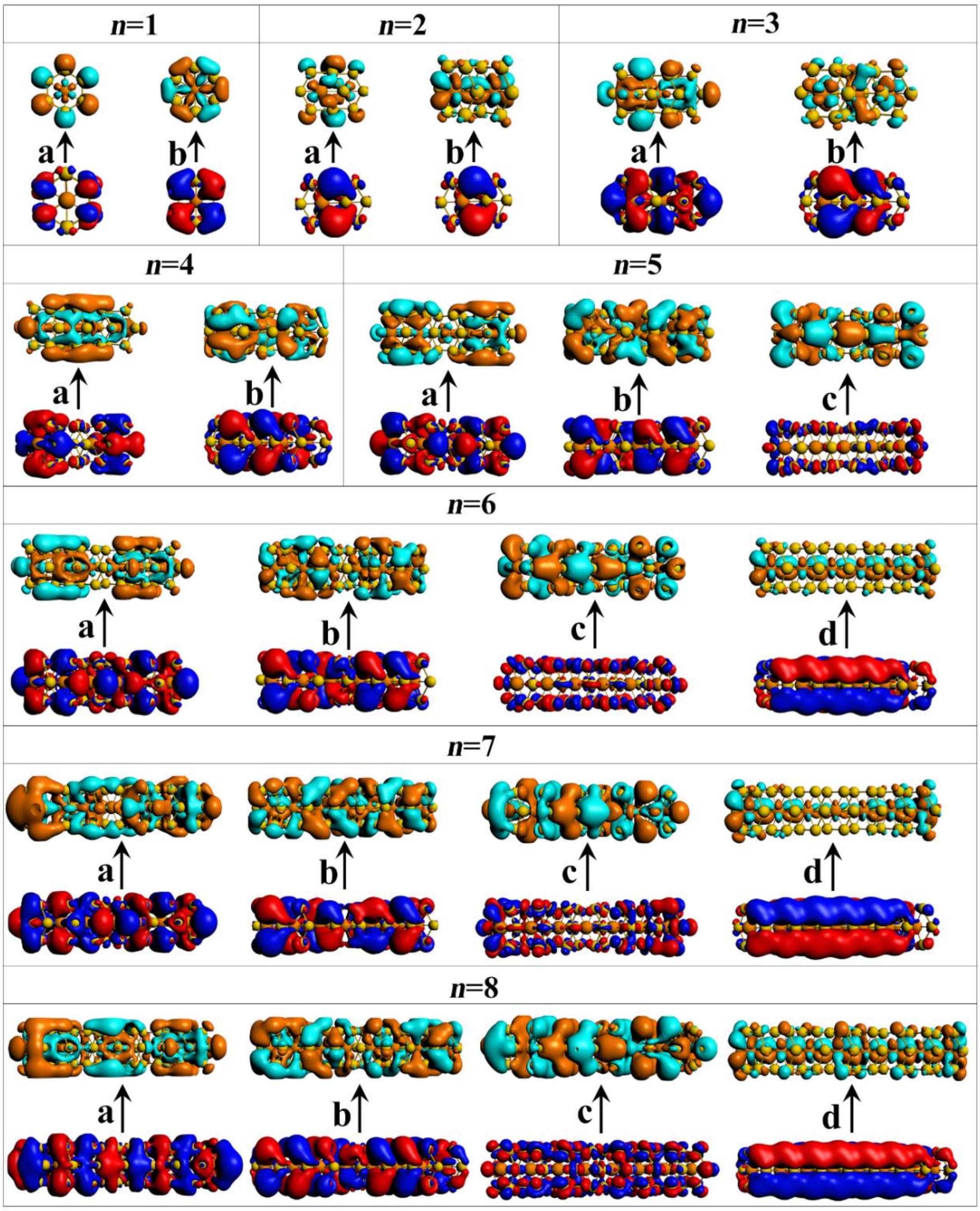

Figure S10. The transition source a-d of $(Th@Au_6)_n$ (n = 1-8) in the UV-Vis absorption spectra. To visualize the contribution of Th atoms more clearly, the orbitals of the transition sources a-d in the manuscript were scaled up equally.

Table S1. Interaction analyses of monomer. The BE and E_{int} between the central atom and the coinage metal rings in the monomer, and average atomization energy (AE) of each atom in the monomer.

	$Th@Au_6$	$Th@Ag_6$	$Th@Cu_6$
BE (eV)	-11.25	-6.22	-8.73
AE (eV)	-2.82	-1.88	-2.37
E_{int} (eV)	-2.99	-2.15	-2.74

We only focus on the binding ability between fragments, so BE formula:

$$\mathrm{BE}[(\mathrm{Th@Au_6})_n] = [\mathrm{E}(\mathrm{Th@Au_6})_n - \mathrm{E}(\mathrm{Th@Au_6}) + \ldots + \mathrm{E}(\mathrm{Th@Au_6})]/(\mathrm{n}-1)$$

Atomization energy (AE) formula:

$$\mathrm{AE}[\mathrm{Th@Au_6}] = [\mathrm{E}(\mathrm{Th@Au_6}) - \mathrm{E}(\mathrm{Th}) + \mathrm{E}(6\mathrm{Au})]/7$$

The total interaction energy of energy decomposition can be divided into four parts, namely Pauli repulsion, electrostatic interaction, orbital interaction and dispersion interaction:

$$\mathrm{E_{int}} = \mathrm{E_{pauli}} + \mathrm{E_{elstat}} + \mathrm{E_{orb}} + \mathrm{E_{dis}}$$

The first term is repulsive interaction, and the last three terms are attractive interaction.

The BE and E_{int} between the central atom and the coinage metal rings in the monomers and the atomization energy (AE) of each atom are calculated. As the shell changes from Au to Cu, the BE between the central atom and coinage metal rings of $Th@Au_6$ is the largest, and $Th@Ag_6$ is the lowest. The same result was obtained in the calculation of E_{int} and AE. This shows that compared with other structures, $Th@Au_6$ is the most stable structure and suitable for assembly.

Table S2. The Wiberg bond order of the central and coinage metal atoms in $Th@M_6$.

	Th-Au	Th-Ag	Th-Cu
Wiberg	1.43	1.16	1.21

The Wiberg bond order was further carried out on the Th-M bonds to explore the bonding strength analysis. It can be seen that as the shell atoms change from Au to Cu, the law of the Wiberg bond order is: Th-Au > Th-Cu > Th-Ag.

Table S3. The charge values of each atom in $Th@M_6$ obtained from the Voronoi deformation

density (VDD) charge distribution analyses (charge in e). The minus sign indicates negative charge.

Th@Au$_6$							
Number/atoms	1 Th	2 Au	3 Au	4 Au	5 Au	6 Au	7 Au
VDD charge (e)	0.553	-0.091	-0.092	-0.092	-0.091	-0.092	-0.092
Th@Ag$_6$							
Number/atoms	1 Th	2 Ag	3 Ag	4 Ag	5 Ag	6 Ag	7 Ag
VDD charge (e)	0.596	-0.100	-0.099	-0.099	-0.100	-0.099	-0.099
Th@Cu$_6$							
Number/atoms	1 Th	2 Cu	3 Cu	4 Cu	5 Cu	6 Cu	7 Cu
VDD charge (e)	0.471	-0.077	-0.079	-0.079	-0.077	-0.079	-0.079

Table S4. The charge values of each atom in (Th@Au$_6$)$_n$ obtained from the Voronoi deformation density (VDD) charge distribution analysis (charge in e). The minus sign indicates negative charge.

(Th@Au$_6$)$_2$							
Number/atoms	1 Th	2 Au	3 Au	4 Au	5 Au	6 Au	7 Au
VDD charge (e)	0.356	-0.071	0.036	-0.071	-0.083	-0.083	-0.083
Number/atoms	8 Au	9 Au	10 Au	11 Au	12 Au	13 Au	14 Au
VDD charge (e)	0.356	-0.071	0.036	-0.071	-0.083	-0.083	-0.083
(Th@Au$_6$)$_3$							
Number/atoms	1 Th	2 Au	3 Au	4 Au	5 Au	6 Au	7 Au
VDD charge (e)	0.127	-0.047	0.039	-0.047	-0.047	0.039	-0.047
Number/atoms	8 Au	9 Au	10 Au	11 Au	12 Au	13 Au	14 Au
VDD charge (e)	0.355	-0.066	0.013	-0.066	-0.080	-0.085	-0.080
Number/atoms	15 Au	16 Au	17 Au	18 Au	19 Au	20 Au	21 Au
VDD charge (e)	0.355	-0.080	-0.066	0.013	-0.066	-0.08	-0.085

$(Th@Au_6)_4$							
Number/atoms	1 Th	2 Au	3 Au	4 Au	5 Au	6 Au	7 Au
VDD charge (e)	0.132	-0.040	0.038	-0.040	-0.042	0.010	-0.042
Number/atoms	8 Au	9 Au	10 Au	11 Au	12 Au	13 Au	14 Au
VDD charge (e)	0.357	-0.066	0.014	-0.066	-0.082	-0.091	-0.082
Number/atoms	15 Au	16 Au	17 Au	18 Au	19 Au	20 Au	21 Au
VDD charge (e)	0.357	-0.066	0.014	-0.066	-0.082	-0.091	-0.082
Number/atoms	22 Au	23 Au	24 Au	25 Au	26 Au	27 Au	28 Au
VDD charge (e)	0.132	-0.040	0.038	-0.04	-0.042	0.010	-0.042

$(Th@Au_6)_5$							
Number/atoms	1 Th	2 Au	3 Au	4 Au	5 Au	6 Au	7 Au
VDD charge (e)	0.136	-0.035	0.010	-0.034	-0.034	0.010	-0.035
Number/atoms	8 Au	9 Au	10 Au	11 Au	12 Au	13 Au	14 Au
VDD charge (e)	0.124	-0.044	0.010	-0.042	-0.040	0.035	-0.044
Number/atoms	15 Au	16 Au	17 Au	18 Au	19 Au	20 Au	21 Au
VDD charge (e)	0.367	-0.070	0.012	-0.068	-0.082	-0.081	-0.081
Number/atoms	22 Au	23 Au	24 Au	25 Au	26 Au	27 Au	28 Au
VDD charge (e)	0.124	-0.043	0.035	-0.044	-0.042	0.012	-0.043
Number/atoms	29 Au	30 Au	31 Au	32 Au	33 Au	34 Au	35 Au
VDD charge (e)	0.365	-0.069	0.013	-0.068	-0.084	-0.085	-0.085

$(Th@Au_6)_6$							
Number/atoms	1 Th	2 Au	3 Au	4 Au	5 Au	6 Au	7 Au
VDD charge (e)	0.126	-0.042	0.010	-0.042	-0.044	0.035	-0.044
Number/atoms	8 Au	9 Au	10 Au	11 Au	12 Au	13 Au	14 Au
VDD charge (e)	0.134	-0.038	0.011	-0.038	-0.037	0.011	-0.037
Number/atoms	15 Au	16 Au	17 Au	18 Au	19 Au	20 Au	21 Au

VDD charge (e)	0.363	-0.083	-0.067	0.015	-0.067	-0.083	-0.083
Number/atoms	22 Au	23 Au	24 Au	25 Au	26 Au	27 Au	28 Au
VDD charge (e)	0.125	-0.043	0.035	-0.043	-0.042	0.011	-0.042
Number/atoms	29 Au	30 Au	31 Au	32 Au	33 Au	34 Au	35 Au
VDD charge (e)	0.363	-0.066	0.016	-0.066	-0.083	-0.084	-0.083
Number/atoms	36 Au	37 Au	38 Au	39 Au	40 Au	41 Au	42 Au
VDD charge (e)	0.134	-0.037	-0.038	0.010	-0.038	-0.037	0.011

Table S5. Electron excitation wavelength, transition orbitals, the contribution of corresponding Th atoms and the contribution of Th valence shell of typical transitions between SAMOs in the UV-Vis spectra of $(Th@Au_6)_n$ (n=1-8). Contributions of Th atoms that account for less than 1% are not included in the statistics. The table only shows that the oscillator strength is relatively large. The H and L refer to HOMO and LUMO, respectively.

motifs	λ (nm)	Transitions (Th contribution)	Contribution of Th atomic valence shell/%
n=1	181.27	H-12 (2.32) → L+11 (21.02)	5f: 1.02, 6d: 1.30 → 5f: 21.02
	185.66	H-21 (18.07) → L+6 (79.54)	6d: 18.07 → 5f: 79.54
	199.45	H-3 (0.00) → L+12 (51.11)	0.00 → 6d: 14.51, 7p:36.60
	216.86	H-18 (7.15) → L+2 (88.32)	6d: 4.90, 7p:2.25 → 7s:21.63, 5f: 64.49, 7p:2.20
	242.38	H-5 (0.00) → L+9 (71.42)	0.00 → 7s:28.74, 6d: 16.92, 7p:25.76
	253.60	H-6 (1.22) → L+8 (44.41)	6d: 1.22 → 5f: 44.41
	260.33	H-12 (2.32) → L+3 (41.20)	5f:1.02, 6d:1.30 → 5f:41.20
	265.16	H-12 (2.32) → L+1 (73.14)	5f:1.02, 6d:1.30 → 5f: 6.36, 6d:66.78
	385.96	H-1 (5.89) → L+2 (88.32)	6d:1.44, 7p:4.45 → 7s: 21.63, 5f: 64.49, 7p:2.20
	461.43	H (14.68) → L+6 (79.54)	6d: 14.68 → 5f: 79.54
n=2	345.88	H-1 (16.22) → L+25 (42.91)	5f: 1.01, 6d: 13.35, 7p: 1.86 → 5f: 15.03, 6d: 25.74, 7p: 2.14
	368.01	H-5 (5.86) → L+6	6d: 1.16, 7p: 4.70 → 7s:6.61, 5f: 42.45, 6d: 13.71,

		(64.23)	7p: 1.46
	473.54	H-2 (19.14) → L+11 (87.35)	7s:2.58, 6d: 16.56 → 5f: 75.05, 6d: 12.30
	475.26	H-1 (16.22) → L+14 (94.01)	5f: 1.01, 6d: 13.35, 7p: 1.86 → 5f: 91.05, 6d: 1.41, 7p: 1.55
	492.59	H-2 (19.14) → L+9 (89.40)	7s:2.58, 6d: 16.56 → 7s: 13.32, 5f: 61.11, 6d: 11.33 7p:3.64
n=3	358.86	H-7 (6.21) → L+18 (94.58)	6d: 2.93, 7p:3.28 → 7s: 7.71, 5f: 75.00, 6d: 11.87
	362.65	H-8 (10.92) → L+11 (72.05%)	7s: 1.31, 6d: 9.61 → 7s: 5.82, 5f: 49.62, 6d: 12.72, 7p:3.89
	363.17	H-4 (20.00) → L+25 (85.82)	6d: 20.00 →5f: 84.35, 6d: 1.47
	377.73	H-2 (21.62) → L+29 (54.73)	7s: 1.34, 6d: 19.20, 7p: 1.08 →7s: 8.89, 5f: 35.44, 6d: 4.04, 7p: 6.27
	501.12	H-2 (21.62) → L+12 (79.75)	7s: 1.34, 6d: 19.20, 7p: 1.08 →7s: 1.01, 5f: 75.16, 6d: 3.58
	509.58	H (16.16) → L+21 (85.92)	5f: 1.48, 6d: 13.66, 7p: 1.02 → 5f: 79.09, 6d: 6.83
n=4	362.76	H-14 (0.00) → L+9 (53.43)	0.00 →5f: 12.99, 6d: 40.44
	370.71	H-3 (22.40) → L+39 (46.46)	6d: 22.40 →7s: 9.06, 5f: 28.25, 6d: 5.02, 7p: 4.13
	377.16	H-20 (5.81) → L+5 (21.36)	7s:4.58, 7p: 1.23 →5f: 21.36
	387.71	H-6 (10.98) → L+29 (57.61)	7s: 1.03, 6d: 9.95 →7s: 30.08, 5f: 8.04, 6d: 14.38, 7p: 5.11
	648.30	H (13.21) → L+17 (69.49)	5f: 2.04, 6d: 11.17 →5f: 53.93, 6d: 15.56
n=5	383.23	H-10 (15.17) → L+20 (71.93)	6d: 15.17 →5f: 71.93
	386.28	H-6 (9.97) → L+38 (47.36)	6d: 9.97 →7s: 26.34, 5f: 10.95, 6d: 10.07
	392.61	H-2 (16.41) → L+46 (68.75)	6d: 16.41 →5f: 64.13, 6d: 3.39, 7p: 1.23
	397.60	H-32 (2.77) → L+2 (35.81)	7s: 2.77 →5f: 1.21, 6d: 34.60
	409.33	H-12 (11.55) → L+10 (45.21)	6d: 11.55 →5f: 12.85, 6d: 10.92, 7p: 21.44
	705.20	H-1 (16.54) → L+8 (50.15)	5f: 1.34, 6d: 15.20 →5f: 10.84, 6d: 37.56, 7p: 1.75
n=6	336.24	H-31 (4.01) → L+13 (57.55)	7s: 4.01 →7s: 5.48, 5f: 23.43, 6d: 24.23, 7p: 4.41

	339.20	H-58 (0.00) → L+3 (34.30%	0.00 →5f: 2.32, 6d: 31.98
	395.06	H-10 (19.85) → L+30 (90.02)	6d: 19.85 →5f: 88.44, 6d: 1.58
	430.36	H-20 (4.5) → L+4 (38.55)	6d: 1.56, 7p:2.94 →7s: 4.51, 5f: 5.52, 6d: 28.52
	442.74	H-3 (16.79) → L+42 (56.71)	6d: 16.79 →7s: 17.31, 5f: 26.98, 6d: 8.17, 7p:4.25
	448.65	H-15 (10.45) → L+8 (21.81)	6d: 10.45 →5f: 16.36, 6d: 5.45
	716.90	H-6 (16.25) → L+17 (57.43)	6d: 16.25 →7s: 5.60, 5f: 8.41, 6d: 41.24, 7p: 2.18
	752.13	H-1 (15.67) → L+17 (59.66)	5f: 1.72, 6d: 13.95 →5f: 27.60, 6d: 32.06
n=7	347.08	H-83 (0.00) → L+3 (31.37)	0.00 →7s: 1.44, 5f: 1.53, 6d: 28.40
	386.64	H-76 (0.00) → L+1 (55.44)	0.00 →7s: 7.85, 6d: 47.59
	388.85	H-3 (19.18) → L+65 (55.87%)	6d: 19.18 →7s: 10.97, 5f: 39.09, 6d: 4.12, 7p: 1.69
	395.29	H-24 (4.34) → L+9 (12.31)	7s: 1.04, 7p: 3.30 →5f: 11.21, 6d: 1.10
	395.59	H-12 (21.80) → L+35 (90.27)	6d: 21.80 →5f: 88.00, 6d: 2.27
	398.23	H-5 (15.62) → L+71 (85.25)	6d: 15.62 →7s: 1.02, 5f: 84.23
	442.16	H-3 (19.18) → L+48 (73.75)	6d: 19.18 →7s: 6.00, 5f: 59.16, 6d: 5.54, 7p:3.05
	452.73	H-11 (13.26) → L+23 (52.71)	6d: 13.26 →7s: 2.67, 5f: 39.54, 6d: 10.50
	734.35	H-5 (15.62) → L+7 (36.82)	6d: 15.62 →7s: 3.08, 5f: 16.57, 6d: 15.41, 7p: 1.76
	782.41	H-2 (14.63) → L+12 (49.85)	5f: 1.03, 6d: 13.60 →5f: 13.09, 6d: 36.76
	798.02	H (10.20) → L+21 (58.10)	5f: 2.26, 6d: 7.76 →5f: 32.95, 6d: 25.15
	868.76	H (10.20) → L+18 (52.36)	5f: 2.26, 6d: 7.76 →5f: 13.25, 6d: 39.11
n=8	361.52	H-88 (0.00) →L+3 (27.11)	0.00 →7s: 2.08, 5f: 1.55, 6d: 23.48
	393.74	H (9.00) →L+106 (57.27)	5f: 1.62, 6d: 6.31, 7p:1.07 →5f: 29.04, 6d: 15.71, 7p:12.52
	396.22	H-14 (21.17) → L+40 (87.04)	6d: 21.17 →5f: 85.49, 6d: 1.55

397.67	H-9 (21.84) → L+56 (58.36)	6d: 21.84 →7s: 15.81, 5f: 33.73, 6d: 6.47, 7p:2.35
399.06	H-1 (13.00) → L+101 (46.6)	5f: 1.43, 6d: 11.57 →5f: 10.01, 6d: 35.56, 7p:1.03
401.65	H-10 (11.75) → L+48 (76.96)	6d: 11.75 →7s:10.99, 5f: 56.81, 6d: 7.76, 7p:1.40
409.31	H-9 (21.84) →L+52 (76.68)	6d: 21.84 →5f: 71.59, 6d: 2.08, 7p:3.01
458.44	H (9.00) → L+79 (41.81)	5f: 1.62, 6d: 6.31, 7p:1.07 →5f: 5.54, 6d: 36.27
755.38	H-5 (15.98) → L+9 (20.73)	6d: 15.98 →5f: 15.56, 6d: 5.17
762.59	H-1 (13.00) → L+24 (59.07)	5f:1.43, 6d: 11.57 →5f: 39.41, 6d: 19.66
781.08	H-10 (11.75) → L+4 (53.69)	6d: 11.75 →7s:7.63, 5f: 4.00, 6d: 42.06
786.33	H (9.00) → L+25 (54.96)	5f: 1.62, 6d: 6.31, 7p:1.07 →5f: 41.58, 6d: 6.61, 7p:6.77
885.96	H-1 (13.00) → L+18 (51.44)	5f: 1.43, 6d: 11.57 →5f: 15.23, 6d: 36.21

Table S6. The optimized Th@Au_6 structural coordinate.

**Structure**	**Number**	**Atom**	**x (Å)**	**y (Å)**	**z (Å)**
**Th@Au_6**	1	Th	0.000000	0.000000	-0.041229
	2	Au	-2.407727	-1.390102	0.645241
	3	Au	-2.407727	1.390102	0.645241
	4	Au	0.000000	2.780204	0.645241
	5	Au	2.407727	1.390102	0.645241
	6	Au	2.407727	-1.390102	0.645241
	7	Au	0.000000	-2.780204	0.645241

Table S7. The optimized Th@Ag_6 structural coordinate.

**Structure**	**Number**	**Atom**	**x (Å)**	**y (Å)**	**z (Å)**
**Th@Ag_6**	1	Th	0.000000	0.000000	-0.284625
	2	Ag	-2.414464	-1.393991	0.685807
	3	Ag	-2.414464	1.393991	0.685807
	4	Ag	0.000000	2.787983	0.685807
	5	Ag	2.414464	1.393991	0.685807
	6	Ag	2.414464	-1.393991	0.685807
	7	Ag	0.000000	-2.787983	0.685807

Table S8. The optimized Th@Cu_6 structural coordinate.

Structure	Number	Atom	x (Å)	y (Å)	z (Å)
**Th@Cu_6**	1	Th	-2.308493	-0.522395	0.000000
	2	Cu	-0.925544	0.519049	2.098058
	3	Cu	-1.243917	-1.884055	2.098049
	4	Cu	-1.396266	-3.084674	0.000000
	5	Cu	-1.243917	-1.884055	-2.098049
	6	Cu	-0.925544	0.519049	-2.098058
	7	Cu	-0.765425	1.721400	0.000000

Table S9. The optimized (Th@Au_6)$_2$ structural coordinate.

Structure	Number	Atom	x (Å)	y (Å)	z (Å)
**(Th@Au_6)$_2$**	1	Th	0.988657	1.835872	0.000000
	2	Au	1.373938	0.372673	2.447246
	3	Au	1.795485	-1.046289	0.000000
	4	Au	1.373938	0.372673	-2.447246
	5	Au	0.154378	2.982472	-2.464340
	6	Au	-0.496149	4.278561	0.000000
	7	Au	0.154378	2.982472	2.464340
	8	Th	-0.988657	-1.835872	0.000000
	9	Au	-1.373938	-0.372673	2.447246
	10	Au	-1.795485	1.046289	0.000000
	11	Au	-1.373938	-0.372673	-2.447246
	12	Au	-0.154378	-2.982472	-2.464340
	13	Au	0.496149	-4.278561	0.000000
	14	Au	-0.154378	-2.982472	2.464340

Table S10. The optimized (Th@Ag_6)$_2$ structural coordinate.

Structure	Number	Atom	x (Å)	y (Å)	z (Å)
**(Th@Ag_6)$_2$**	1	Th	-2.577255	0.456096	-0.725100
	2	Ag	-1.927882	3.759245	-1.308639
	3	Ag	0.303747	2.143404	-0.575310
	4	Ag	0.382413	-0.587524	-1.094583
	5	Ag	-1.637568	-2.472566	-0.772793
	6	Ag	-4.287820	-2.068670	-1.381282
	7	Ag	-5.685924	0.312251	-1.233862
	8	Th	-1.001673	1.704130	-3.389117
	9	Ag	-4.198710	2.427798	-2.426002
	10	Ag	-3.774656	-0.155223	-3.555180
	11	Ag	-1.312821	-1.440738	-3.411235
	12	Ag	1.225690	-0.375124	-3.809971

	13	Ag	2.059817	2.093363	-2.934514
	14	Ag	0.514049	4.366804	-2.641440

Table S11. The optimized $(Th@Cu_6)_2$ structural coordinate.

Structure	Number	Atom	x (Å)	y (Å)	z (Å)
**$(Th@Cu_6)_2$**	1	Th	-2.428833	-0.528493	0.000000
	2	Cu	-1.109772	0.555350	2.152903
	3	Cu	-1.072478	-1.855613	1.994978
	4	Cu	-1.498114	-3.135692	0.000000
	5	Cu	-1.072478	-1.855613	-1.994978
	6	Cu	-1.109772	0.555350	-2.152903
	7	Cu	-0.517659	1.649031	0.000000
	8	Th	2.428833	0.528493	0.000000
	9	Cu	1.072478	1.855613	-1.994978
	10	Cu	1.109772	-0.555350	-2.152903
	11	Cu	0.517659	-1.649031	0.000000
	12	Cu	1.109772	-0.555350	2.152903
	13	Cu	1.072478	1.855613	1.994978
	14	Cu	1.498114	3.135692	0.000000

Table S12. The optimized $(Th@Au_6)_3$ structural coordinate.

Structure	Number	Atom	x (Å)	y (Å)	z (Å)
**$(Th@Au_6)_3$**	1	Th	0.000000	0.000000	0.000000
	2	Au	-0.874404	1.168765	2.481502
	3	Au	-1.564194	2.479987	0.000000
	4	Au	-0.874404	1.168765	-2.481502
	5	Au	0.874404	-1.168765	-2.481502
	6	Au	1.564194	-2.479987	0.000000
	7	Au	0.874404	-1.168765	2.481502
	8	Th	1.029387	3.812324	0.000000
	9	Au	1.716107	2.420507	2.430371
	10	Au	2.621966	1.210816	0.000000
	11	Au	1.716107	2.420507	-2.430371
	12	Au	-0.007410	4.732344	-2.472958
	13	Au	-0.914875	5.895336	0.000000
	14	Au	-0.007410	4.732344	2.472958
	15	Th	-1.029387	-3.812324	0.000000
	16	Au	0.007410	-4.732344	-2.472958
	17	Au	-1.716107	-2.420507	-2.430371
	18	Au	-2.621966	-1.210816	0.000000
	19	Au	-1.716107	-2.420507	2.430371

	20	Au	0.007410	-4.732344	2.472958
	21	Au	0.914875	-5.895336	0.000000

Table S13. The optimized $(Th@Au_6)_4$ structural coordinate.

Structure	Number	Atom	x (Å)	y (Å)	z (Å)
**$(Th@Au_6)_4$**	1	Th	-0.001571	1.860784	0.000000
	2	Au	-1.127677	2.737580	2.493155
	3	Au	-2.160737	3.828230	0.000000
	4	Au	-1.127677	2.737580	-2.493155
	5	Au	1.143543	0.911274	-2.466541
	6	Au	2.293038	-0.049105	0.000000
	7	Au	1.143543	0.911274	2.466541
	8	Th	-0.029608	5.818033	0.000000
	9	Au	1.011749	4.650991	2.426839
	10	Au	2.192895	3.730083	0.000000
	11	Au	1.011749	4.650991	-2.426839
	12	Au	-1.273021	6.402454	-2.480351
	13	Au	-2.469094	7.283749	0.000000
	14	Au	-1.273021	6.402454	2.480351
	15	Th	0.029608	-5.818033	0.000000
	16	Au	-1.011749	-4.650991	2.426839
	17	Au	-2.192895	-3.730083	0.000000
	18	Au	-1.011749	-4.650991	-2.426839
	19	Au	1.273021	-6.402454	-2.480351
	20	Au	2.469094	-7.283749	0.000000
	21	Au	1.273021	-6.402454	2.480351
	22	Th	0.001571	-1.860784	0.000000
	23	Au	1.127677	-2.737580	2.493155
	24	Au	2.160737	-3.828230	0.000000
	25	Au	1.127677	-2.737580	-2.493155
	26	Au	-1.143543	-0.911274	-2.466541
	27	Au	-2.293038	0.049105	0.000000
	28	Au	-1.143543	-0.911274	2.466541

Table S14. The optimized $(Th@Au_6)_5$ structural coordinate.

Structure	Number	Atom	x (Å)	y (Å)	z (Å)
**$(Th@Au_6)_5$**	1	Th	-0.287753	-3.719930	0.000000
	2	Au	-1.365957	-2.674712	2.461613
	3	Au	-2.417507	-1.631591	0.000000
	4	Au	-1.365957	-2.674712	-2.461613
	5	Au	0.768876	-4.660540	-2.499937
	6	Au	1.718098	-5.838566	0.000000

7	Au	0.768876	-4.660540	2.499937
8	Th	0.000000	0.000000	0.000000
9	Au	1.037053	-1.023184	2.475880
10	Au	2.125357	-2.083186	0.000000
11	Au	1.037053	-1.023184	-2.475880
12	Au	-1.037053	1.023184	-2.475880
13	Au	-2.125357	2.083186	0.000000
14	Au	-1.037053	1.023184	2.475880
15	Th	-0.555804	-7.666328	0.000000
16	Au	0.641769	-8.329682	-2.483098
17	Au	-1.502641	-6.414686	-2.424644
18	Au	-2.618816	-5.415498	0.000000
19	Au	-1.502641	-6.414686	2.424644
20	Au	0.641769	-8.329682	2.483098
21	Au	1.779223	-9.292845	0.000000
22	Th	0.555804	7.666328	0.000000
23	Au	-0.641769	8.329682	2.483098
24	Au	-1.779223	9.292845	0.000000
25	Au	-0.641769	8.329682	-2.483098
26	Au	1.502641	6.414686	-2.424644
27	Au	2.618816	5.415498	0.000000
28	Au	1.502641	6.414686	2.424644
29	Th	0.287753	3.719930	0.000000
30	Au	1.365957	2.674712	-2.461613
31	Au	-0.768876	4.660540	-2.499937
32	Au	-1.718098	5.838566	0.000000
33	Au	-0.768876	4.660540	2.499937
34	Au	1.365957	2.674712	2.461613
35	Au	2.417507	1.631591	0.000000

Table S15. The optimized $(Th@Au_6)_6$ structural coordinate.

Structure	Number	Atom	x (Å)	y (Å)	z (Å)
**$(Th@Au_6)_6$**	1	Th	-1.365356	-5.434802	0.000000
	2	Au	-2.242891	-4.218979	2.461734
	3	Au	-3.113735	-3.016957	0.000000
	4	Au	-2.242891	-4.218979	-2.461734
	5	Au	-0.465096	-6.527567	-2.500619
	6	Au	0.281974	-7.845631	0.000000
	7	Au	-0.465096	-6.527567	2.500619
	8	Th	-0.457287	-1.814408	0.000000
	9	Au	0.395310	-2.977384	2.483170
	10	Au	1.286809	-4.218879	0.000000
	11	Au	0.395310	-2.977384	-2.483170

	12	Au	-1.316831	-0.618790	-2.469272
	13	Au	-2.189092	0.603252	0.000000
	14	Au	-1.316831	-0.618790	2.469272
	15	Th	-2.254483	-9.280998	0.000000
	16	Au	-1.189503	-10.129849	-2.485959
	17	Au	-2.991545	-7.889705	-2.423075
	18	Au	-3.939084	-6.728998	0.000000
	19	Au	-2.991545	-7.889705	2.423075
	20	Au	-1.189503	-10.129849	2.485959
	21	Au	-0.223758	-11.271983	0.000000
	22	Th	1.365356	5.434802	0.000000
	23	Au	0.465096	6.527567	2.500619
	24	Au	-0.281974	7.845631	0.000000
	25	Au	0.465096	6.527567	-2.500619
	26	Au	2.242891	4.218979	-2.461734
	27	Au	3.113735	3.016957	0.000000
	28	Au	2.242891	4.218979	2.461734
	29	Th	2.254483	9.280998	0.000000
	30	Au	2.991545	7.889705	2.423075
	31	Au	3.939084	6.728998	0.000000
	32	Au	2.991545	7.889705	-2.423075
	33	Au	1.189503	10.129849	-2.485959
	34	Au	0.223758	11.271983	0.000000
	35	Au	1.189503	10.129849	2.485959
	36	Th	0.457287	1.814408	0.000000
	37	Au	1.316831	0.618790	-2.469272
	38	Au	-0.395310	2.977384	-2.483170
	39	Au	-1.286809	4.218879	0.000000
	40	Au	-0.395310	2.977384	2.483170
	41	Au	1.316831	0.618790	2.469272
	42	Au	2.189092	-0.603252	0.000000

Table S16. The optimized $(Th@Au_6)_7$ structural coordinate.

Structure	Number	Atom	x (Å)	y (Å)	z (Å)
**$(Th@Au_6)_7$**	1	Th	-0.303150	-7.465618	0.000000
	2	Au	-1.411580	-6.450434	2.460177
	3	Au	-2.517511	-5.461264	0.000000
	4	Au	-1.411580	-6.450434	-2.460177
	5	Au	0.798692	-8.347422	-2.502595
	6	Au	1.798546	-9.489415	0.000000
	7	Au	0.798692	-8.347422	2.502595
	8	Th	-0.165131	-3.740033	0.000000
	9	Au	0.915156	-4.694352	2.483574

	10	Au	2.041136	-5.731115	0.000000
	11	Au	0.915156	-4.694352	-2.483574
	12	Au	-1.239076	-2.734220	-2.469037
	13	Au	-2.347954	-1.719361	0.000000
	14	Au	-1.239076	-2.734220	2.469037
	15	Th	-0.391435	-11.414043	0.000000
	16	Au	0.830184	-12.022538	-2.484673
	17	Au	-1.392312	-10.199618	-2.422955
	18	Au	-2.558342	-9.258939	0.000000
	19	Au	-1.392312	-10.199618	2.422955
	20	Au	0.830184	-12.022538	2.484673
	21	Au	2.007074	-12.943471	0.000000
	22	Th	0.000000	0.000000	0.000000
	23	Au	1.080342	-0.979146	2.475874
	24	Au	2.179745	-2.014592	0.000000
	25	Au	1.080342	-0.979146	-2.475874
	26	Au	-1.080342	0.979146	-2.475874
	27	Au	-2.179745	2.014592	0.000000
	28	Au	-1.080342	0.979146	2.475874
	29	Th	0.303150	7.465618	0.000000
	30	Au	-0.798692	8.347422	2.502595
	31	Au	-1.798546	9.489415	0.000000
	32	Au	-0.798692	8.347422	-2.502595
	33	Au	1.411580	6.450434	-2.460177
	34	Au	2.517511	5.461264	0.000000
	35	Au	1.411580	6.450434	2.460177
	36	Th	0.391435	11.414043	0.000000
	37	Au	1.392312	10.199618	2.422955
	38	Au	2.558342	9.258939	0.000000
	39	Au	1.392312	10.199618	-2.422955
	40	Au	-0.830184	12.022538	-2.484673
	41	Au	-2.007074	12.943471	0.000000
	42	Au	-0.830184	12.022538	2.484673
	43	Th	0.165131	3.740033	0.000000
	44	Au	1.239076	2.734220	-2.469037
	45	Au	-0.915156	4.694352	-2.483574
	46	Au	-2.041136	5.731115	0.000000
	47	Au	-0.915156	4.694352	2.483574
	48	Au	1.239076	2.734220	2.469037
	49	Au	2.347954	1.719361	0.000000

Table S17. The optimized $(Th@Au_6)_8$ structural coordinate.

Structure	Number	Atom	x (Å)	y (Å)	z (Å)

**(Th@Au$_6$)$_8$**	1	Th	-0.290201	-9.333733	0.000000
	2	Au	-1.411348	-8.330127	2.459653
	3	Au	-2.528022	-7.355333	0.000000
	4	Au	-1.411348	-8.330127	-2.459653
	5	Au	0.821638	-10.201050	-2.503327
	6	Au	1.836060	-11.331429	0.000000
	7	Au	0.821638	-10.201050	2.503327
	8	Th	-0.194892	-5.607586	0.000000
	9	Au	0.894096	-6.547529	2.484933
	10	Au	2.033676	-7.573253	0.000000
	11	Au	0.894096	-6.547529	-2.484933
	12	Au	-1.278815	-4.607956	-2.467582
	13	Au	-2.404983	-3.611965	0.000000
	14	Au	-1.278815	-4.607956	2.467582
	15	Th	-0.329324	-13.282950	0.000000
	16	Au	0.900210	-13.875740	-2.484684
	17	Au	-1.345288	-12.080710	-2.422805
	18	Au	-2.522948	-11.154629	0.000000
	19	Au	-1.345288	-12.080710	2.422805
	20	Au	0.900210	-13.875740	2.484684
	21	Au	2.088489	-14.781977	0.000000
	22	Th	-0.074486	-1.871319	0.000000
	23	Au	1.023618	-2.830281	-2.476462
	24	Au	-1.149923	-0.890090	-2.476093
	25	Au	-2.262310	0.134578	0.000000
	26	Au	-1.149923	-0.890090	2.476093
	27	Au	1.023618	-2.830281	2.476462
	28	Au	2.131592	-3.859183	0.000000
	29	Th	0.074486	1.871319	0.000000
	30	Au	1.149923	0.890090	2.476093
	31	Au	2.262310	-0.134578	0.000000
	32	Au	1.149923	0.890090	-2.476093
	33	Au	-1.023618	2.830281	-2.476462
	34	Au	-2.131592	3.859183	0.000000
	35	Au	-1.023618	2.830281	2.476462
	36	Th	0.290201	9.333733	0.000000
	37	Au	-0.821638	10.201050	2.503327
	38	Au	-1.836060	11.331429	0.000000
	39	Au	-0.821638	10.201050	-2.503327
	40	Au	1.411348	8.330127	-2.459653
	41	Au	2.528022	7.355333	0.000000
	42	Au	1.411348	8.330127	2.459653
	43	Th	0.329324	13.282950	0.000000
	44	Au	1.345288	12.080710	2.422805

45	Au	2.522948	11.154629	0.000000
46	Au	1.345288	12.080710	-2.422805
47	Au	-0.900210	13.875740	-2.484684
48	Au	-2.088489	14.781977	0.000000
49	Au	-0.900210	13.875740	2.484684
50	Th	0.194892	5.607586	0.000000
51	Au	1.278815	4.607956	-2.467582
52	Au	-0.894096	6.547529	-2.484933
53	Au	-2.033676	7.573253	0.000000
54	Au	-0.894096	6.547529	2.484933
55	Au	1.278815	4.607956	2.467582
56	Au	2.404983	3.611965	0.000000

Reference

[1] a) A. Cheng, J. Wang, Q. Zhang, Y. Zhu, Z. Wang, *Chem. Phys. Lett.* **2020**, *752*, 137574; b) C. Thierfelder, P. Schwerdtfeger, A. Koers, A. Borschevsky, B. Fricke, *Phys. Rev. A* **2009**, *80*.

[2] D. R. Lide, *Handbook of Chem and Phys* **1992**.

[3] X. Wu, Z. Qin, H. Xie, R. Cong, X. Wu, Z. Tang, H. Fan, *J. Phys. Chem. A* **2010**, *114*, 12839.

[4] G. M. Whitesides, M. Boncheva, *Proc. Natl. Acad. Sci. U S A* **2002**, *99*, 4769.
